# The prevalence of subdural blood products in extremely premature infants with no history of abusive head trauma, studied by magnetic resonance imaging around term-equivalent age

**DOI:** 10.1007/s00247-024-06060-x

**Published:** 2024-10-14

**Authors:** Maria Olsen Fossmark, Hannah Bakøy, Nils Thomas Songstad, Thorsten Köhler, Derk Avenarius, Stein Magnus Aukland, Karen Rosendahl

**Affiliations:** 1 Department of Clinical Medicine, UiT The Arctic University of Norway, Post Office Box 6050 Langnes, 9037 Tromsø, Norway; 2 Department of Radiology, University Hospital of North Norway, Tromsø, Norway; 3 Department of Paediatrics, Haukeland University Hospital, Bergen, Norway; 4 Department of Paediatrics and Adolescent Medicine, University Hospital of North Norway, Tromsø, Norway; 5 Evidia Norway AS, Tromsø, Norway; 6 Department of Clinical Medicine, University of Bergen, Bergen, Norway; 7 Department of Radiology, Haukeland University Hospital, Bergen, Norway

**Keywords:** Hematoma, Subdural, Infant, Extremely premature, Intracranial haemorrhages, Magnetic resonance imaging, Subarachnoid space

## Abstract

**Background:**

Prematurity and enlarged subarachnoid spaces are both hypothesised to represent an increased risk of subdural haemorrhages (SDHs) in infancy, both with and without a history of abuse.

**Objective:**

To examine the prevalence of a previous haemorrhage, particularly SDHs, in infants born extremely prematurely around term-equivalent age; to examine intra- and inter-observer agreement for identification of haemorrhages; and to examine the width of the subarachnoid spaces.

**Materials and methods:**

A total of 121 extremely premature infants had cerebral magnetic resonance imaging (MRI) performed around term-equivalent age (mean chronological age 14.7 weeks, range 10.3–24.0 weeks). There were no infants investigated for abuse in our cohort. Intracranial haemorrhages were classified as isolated germinal matrix-haemorrhages, parenchymal haemorrhages (cerebellar- and cerebral haemorrhages), or extra-axial haemorrhages (subarachnoid haemorrhages, SDHs, or epidural haemorrhages). Sinocortical width and interhemispheric distance were measured.

**Results:**

No appreciable SDH was detected with the performed sequences. Haemorrhage/blood products related to prematurity were seen in 60 (49.5%) of the neonates. Agreement was good to very good for identification of haemorrhage. The mean sinocortical width was 3.5 mm with a standard deviation (SD) of 1.4 mm on the right side and 3.3 mm (SD 1.2 mm) on the left side. The mean interhemispheric distance was 3.1 mm (SD 1.1 mm). 61.1% of the infants had a sinocortical width > 3 mm on one or both sides.

**Conclusion:**

Our study does not support the hypothesis that premature infants are more prone to SDH unrelated to abusive head trauma during the first 3–4 months of life. A large percentage of the ex-premature infants had prominent subarachnoid spaces.

**Graphical Abstract:**

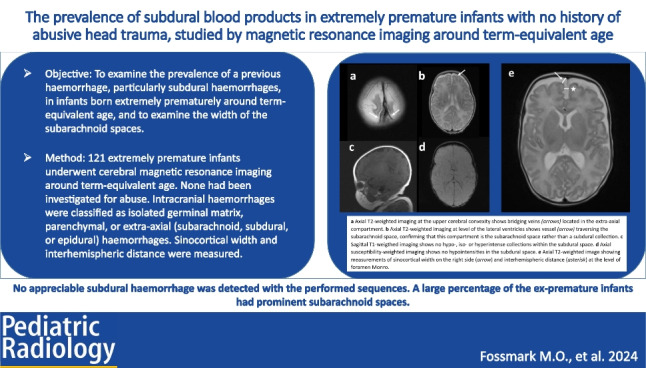

## Background

Infants born prematurely (prior to 37 gestational weeks) and infants with low birth weight (below 2,500 g) are more likely to suffer from maltreatment compared to infants born at term [1–7]. Compared to infants born at term, prematures have a higher risk of being readmitted to the hospital within their first year of life for injuries associated with physical abuse and neglect [2, 4].

Subdural haemorrhages (SDHs) are significantly associated with abusive head trauma (AHT) [8, 9]. However, there is also a high prevalence of SDH in asymptomatic newborns, classified as birth-related SDH, which has been reported at a varied prevalence ranging from 8–45% [10–13]. Asymptomatic SDHs in newborns are typically under 3 mm wide and do not progress to chronic subdural collections [12]. They typically resolve within 4 weeks of age [12, 13] and are completely resolved by 3 months of age [12].

The literature on SDH in premature- and ex-premature children is very sparse, with one study reporting an increased risk of SDH in premature children both with and without a history of abuse [14]. As infants born prematurely are more likely to experience physical abuse and neglect compared to infants born at term, it is of great importance to acquire a comprehensive understanding of the typical brain findings in this population, also for medico-legal reasons.

Several studies suggest that prominent subarachnoid spaces in infants with benign enlargement of the subarachnoid space (BESS) predispose to spontaneous SDHs or SDHs after minor head trauma [15–19]. Compared to infants born at term [20–22], a larger percentage of infants born prematurely have prominent subarachnoid spaces at term-equivalent age [23–25], which may be due to various encephalopathies [23, 24, 26–29]. Libicher and Tröger have proposed upper limits of 3 mm for sinocortical width and 6 mm for interhemispheric distance to distinguish normal from pathologically dilated subarachnoid spaces in infants, as measured on cranial ultrasound [21]. The pathophysiology behind enlarged subarachnoid spaces in ex-premature infants might differ from that of BESS. However, some similarities might exist, such as overstretching of the extra-axial blood vessels, which has been demonstrated by a mathematical model [30]. The association between a greater depth of the subarachnoid space and the increased prevalence of such collections is controversial, though [15, 31].

We aimed to examine the prevalence of a previous intracranial haemorrhage in ex-premature infants close to term-equivalent age with emphasis on SDH, and to examine intra- and inter-observer agreement for identification of haemorrhage. Furthermore, we report the width of the subarachnoid space, measured as sinocortical width and interhemispheric distance on axial magnetic resonance imaging (MRI).

## Material and methods

The study was part of a larger, prospective, population-based cohort study (Baby-PEP; project extreme prematurity) [32]. All women with threatening preterm delivery before 28 weeks of gestation, residing in the defined region of Western Norway (Helse Vest) from 2010 to 2018 and receiving care at Haukeland University Hospital, were invited to participate in Baby-PEP. Within Baby-PEP, attempts were made to perform a brain MRI at term-equivalent age, in addition to an extensive clinical and laboratory follow-up. The inclusion criteria for the current study were infants born at a gestational age less than 28 weeks at Haukeland University Hospital between October 1, 2010, and December 31, 2018, and a consent to participate in the study. This resulted in a total of 178 participants. Of these, four participants withdrew from the study, and one was excluded due to incomplete data. Amongst the 173 participants with available results, some MRI data were missing, due to either early death, transfer to another hospital, or incomplete referral. Participants with missing MRI data were excluded, leaving 121 extremely premature neonates for the present study. There were no infants investigated for abuse in our cohort. Of the included infants, 111 had a cerebral MRI performed at term-equivalent age, defined as a postmenstrual age of 40 weeks ± 2 weeks, with a mean chronological age of 14.3 weeks and a range of 11.8–18.0 weeks at the time of MRI. Of the remaining ten, one infant had an MRI performed at a postmenstrual age of 37.7 weeks. The remaining nine were slightly older, with postmenstrual age ranging from 42.7–49.2 weeks. Of all the included 121 infants, the mean chronological age at the time of MRI was 14.7 weeks, with a range of 10.3–24.0 weeks. Sixty-two (51.2%) of the infants were born by caesarean section. Twenty-three (19.0%) were born vaginally with pre-labour contractions, whereas 36 (29.7%) were born vaginally without pre-labour contractions.

The MRI examinations were performed without sedation, using a “feed-and-wrap”-technique with a 1.5-T MAGNETOM machine (Siemens Healthineers, Erlangen, Germany) and a head coil. The a priori set protocol consisted of an axial T2 turbo spin echo (TSE) or equivalent, sagittal T1 spin echo (SE) or equivalent, axial T1 true inversion recovery (IR), axial diffusion-weighted imaging (DWI) with an apparent diffusion coefficient (ADC) map, and axial susceptibility-weighted imaging (SWI). Technical parameters are listed in Table 1. Susceptibility-weighted imaging (SWI), which is especially sensitive for blood-products, was performed in 98 out of 121 cases. Coronal fluid-attenuated inversion recovery (FLAIR) sequence, which was not part of the a priori set protocol, was performed in 10 out of the 121 infants.

**Table 1:** Technical parameters of magnetic resonance imaging protocol

Sequence	Acquired voxel size (mm^3^)	Slice thickness (mm)	Slice gap (mm)	Repetition time (ms)	Echo time (ms)	Scan time (minutes)
Axial T2 turbo spin echo^a^	0.8 × 0.8 × 4.0	4.0	0.4	4,000.0	118.0	2:14
Sagittal T1 spin echo^b^	0.4 × 0.4 × 4.0	4.0	0.4	450.0	8.4	2:57
Axial T1 true inversion recovery	0.8 × 0.8 × 4.0	4.0	0.4	4,500.0	57.0	2:38
Axial diffusion-weighted imaging	1.2 × 1.2 × 4.0	4.0	0.4	4,800.0	96.0	2:30
Axial susceptibility-weighted imaging	0.8 × 0.8 × 2.0	2.0	0.4	49.0	40.0	3:49

^a,b^The sequences were adjusted using sequences with motion correction or rapid sequences when necessary

Coronal fluid-attenuated inversion recovery sequence was performed in 10 out of the 121 neonates

All MRI examinations were read by two experienced paediatric radiologists with 20 years (S.M.A.) and 8 years (T.K.) of experience in paediatric neuroimaging, independently and masked to other information and findings. For intra-observer agreement, one observer (T.K.) re-read the images after an interval of at least 3 weeks. Haemorrhage/blood products were assessed for presence and localisation, and classified as either extra-axial, germinal matrix-intraventricular, or parenchymal (including both cerebellar- and cerebral haemorrhages/blood products, as well as sequelae of porencephalic cysts). This was followed by measurements of the subarachnoid space (sinocortical width on right and left sides and interhemispheric distance), performed by both S.M.A. and T.K. One independent observer (S.M.A.) measured the subarachnoid spaces, including the sinocortical width on the right and left sides and the interhemispheric distance, on the ten infants who had undergone coronal FLAIR sequences.

### Assessment of extra-axial haemorrhage or blood products

**Fig. 1:**
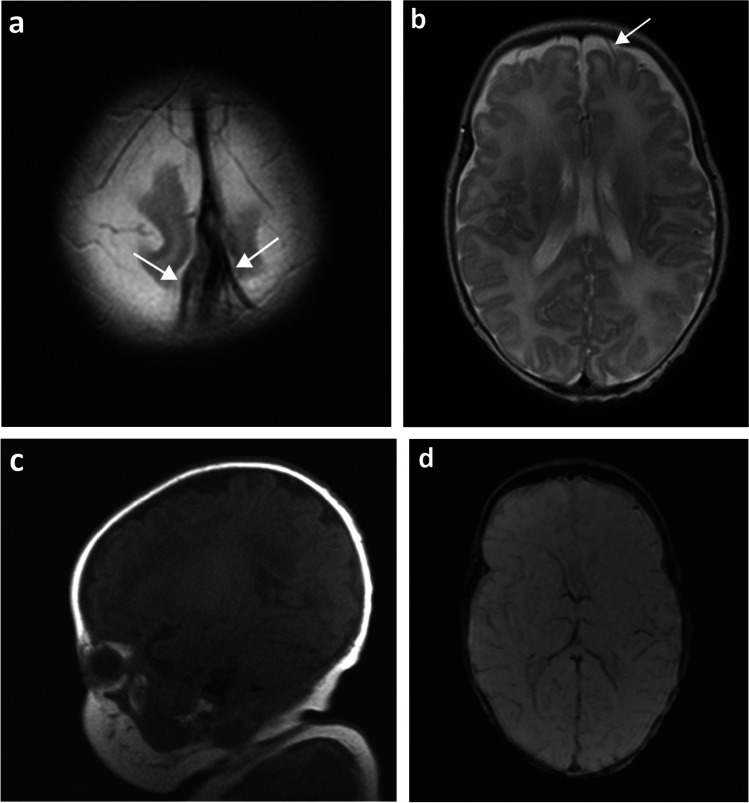
A 16-week-old boy born extremely premature at 25 gestational weeks underwent magnetic resonance imaging at term-equivalent age to assess for subdural haemorrhage. **a** Axial T2-weighted image at the upper cerebral convexity shows bridging veins (*arrows*) located in the extra-axial compartment, draining into the superior sagittal sinus. **b** Axial T2-weighted image at the level of the lateral ventricles shows the vessel (*arrow*) traversing the subarachnoid space, confirming that this compartment is the subarachnoid space rather than a subdural collection. **c** Sagittal T1-weigthed image shows no hypo-, iso-, or hyperintense collections relative to adjacent cortex within the subdural space, suggesting the absence of subdural haemorrhage. **d** Axial susceptibility-weighted image shows no hypointensities in the subdural space, suggesting the absence of subdural haemorrhage

For assessment of extra-axial haemorrhage (subarachnoid haemorrhages, SDHs, and epidural haemorrhages), T1-, T2-, and susceptibility-weighted imaging (SWI) was used. Extra-axial haemorrhages/blood products were defined as collections within either the subarachnoid-, subdural-, or epidural space that could be either hypo-, iso-, or hyperintense on T1- and T2-weighted imaging relative to the adjacent cortex, as the MRI signal intensity of an intracranial haemorrhage may vary due to various factors including the age of the haemorrhage [33–35]. The evaluation of SDH involved assessing the position of bridging veins relative to the cerebral cortical surface; bridging veins located within the extra-axial compartment indicated that the compartment was within the subarachnoid space, thereby excluding the presence of a subdural collection (Fig. 1). Thrombosis or rupture of bridging veins was looked for [36]. On SWI, haemorrhage or blood products were identified as hypointensities caused by susceptibility-related signal loss [33].

### Assessment of germinal matrix-intraventricular haemorrhage or blood products, and parenchymal haemorrhages or blood products

**Fig. 2:**
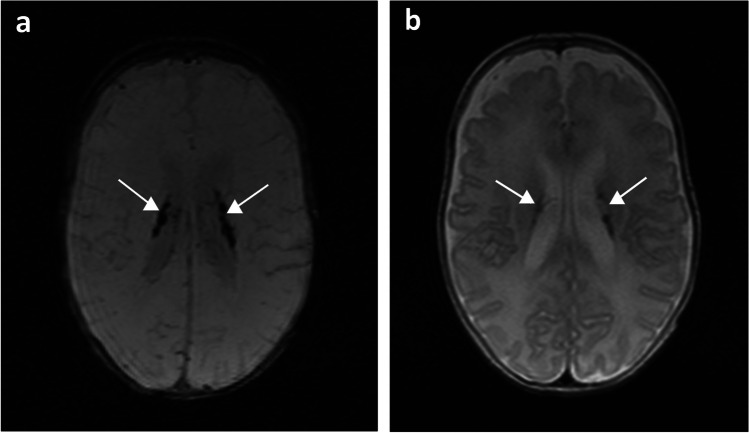
A 13-week-old girl born extremely premature at 27 gestational weeks with magnetic resonance imaging performed at term-equivalent age. **a** Axial susceptibility-weighted image shows bilateral germinal matrix haemorrhages (*arrows*) with susceptibility-related signal loss. **b** Axial T2-weighted image shows bilateral germinal matrix haemorrhages (*arrows*) as hypointensities

**Fig. 3:**
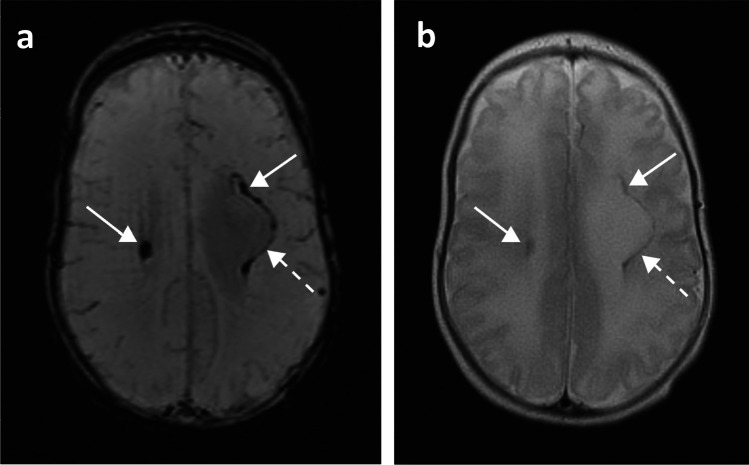
A 14-week-old boy born extremely premature at 26 gestational weeks with magnetic resonance imaging performed at term-equivalent age. **a** Axial susceptibility-weighted image shows bilateral germinal matrix haemorrhages (*arrows*) with susceptibility-related signal loss, and sequelae of a broad-based porencephalic cyst on the left side (*broken arrow*) with adjacent susceptibility-related signal loss. **b** Axial T2-weighted image shows bilateral germinal matrix haemorrhages as hypointensities (*arrows*) and sequelae of a broad-based porencephalic cyst on the left side (*broken arrow*) with adjacent hypointensities

**Fig. 4:**
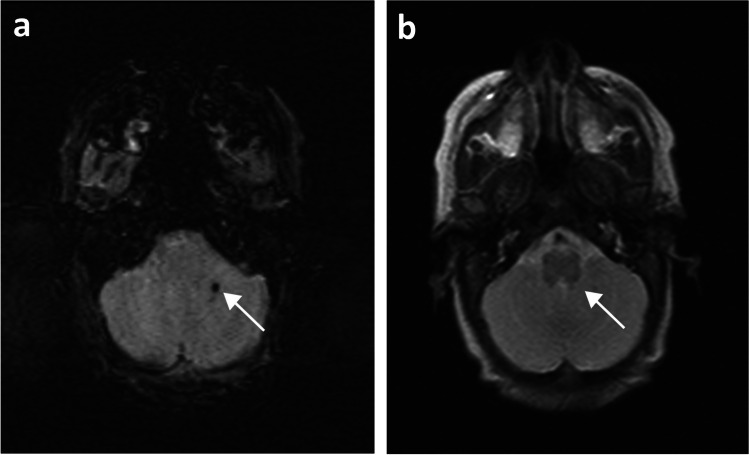
A 17-week-old girl born extremely premature at 23 gestational weeks with magnetic resonance imaging performed at term-equivalent age. **a** Axial susceptibility-weighted image shows a small cerebellar haemorrhage in the left hemisphere (*arrow*) with susceptibility-related signal loss. **b** Axial T2-weighted image shows cerebellar haemorrhage in the left hemisphere as a small hypointensity (*arrow*)

Isolated germinal matrix-intraventricular haemorrhages were defined as susceptibility-related signal loss in the subependymal regions on SWI and hypointensities on T2-weighted images (Fig. 2). Germinal matrix-intraventricular haemorrhages with sequelae of porencephalic cysts were defined as a loss of periventricular white matter, adjacent susceptibility-related signal loss on SWI, and/or hypointensities on T2-weighted images (Fig. 3). Cerebellar haemorrhages were defined as susceptibility-related signal loss in the cerebellum on SWI and/or hypointensities on T2-weighted images (Fig. 4).

### Measurement of the subarachnoid space

**Fig. 5:**
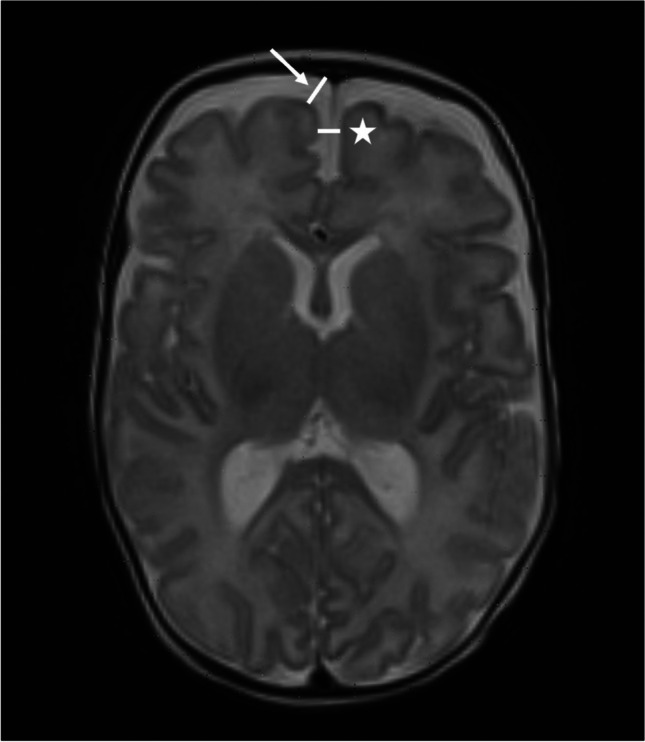
A 13-week-old girl born extremely premature at 27 gestational weeks with magnetic resonance imaging performed at term-equivalent age. Axial T2-weighted image shows measurements of sinocortical width on the right side (*arrow*) and interhemispheric distance (*asterisk*) at the level of foramen of Monro

Sinocortical width (right and left) and interhemispheric distance were measured on an axial T2-weighted image, at the level of foramen of Monro (Fig. 5). On the ten infants who had coronal FLAIR sequences performed, the subarachnoid spaces, including the sinocortical width on the right and left sides and the interhemispheric distance, were measured at the level of foramen of Monro, with the observer blinded to the axial measurement results.

### Statistical analysis

Continuous data were presented as means ± standard deviation (SD) and/or medians (range) as appropriate, ordinal data as medians (ranges), and dichotomous data as proportions. Intra- and inter-observer agreement was analysed using an unweighted Cohen’s kappa coefficient [37] with 95% confidence interval (CI). A kappa score of < 0.2 was considered poor, 0.21–0.40 fair, 0.41–0.60 moderate, 0.61–0.80 good, and 0.81–1.00 very good [38]. Absolute agreement was reported as proportions. All statistical analysis was performed using IBM SPSS version 28 (IBM, Chicago, IL).

## Ethics approval

The study was approved by the Regional Ethics Committee of Western Norway (REK number 15573). Informed consent was given by the carer(s). Data was collected and stored according to the General Data Protection Regulation. The study was performed in accordance with the ethical standards as laid down in the 1964 Declaration of Helsinki.

## Results

A total of 121 MRIs around term-equivalent age of 121 ex-premature infants (57 male) were examined. The demographic data on these 121 infants at the time of MRI examination, along with their chronological age and postmenstrual age at the time of MRI examination, is presented in Table 2.

**Table 2:** Demographic data on 121 neonates born extremely premature at time of magnetic resonance imaging examination around term-equivalent age

Parameters	Value
Total number (male)	121 (57)
Mean gestational age, weeks (SD)	26.2 (1.2)
Range of gestational age, weeks	23.0–27.8
Mean birth weight, grams (SD)	839.0 (205.0)
Range of birth weight, grams	444.0–1470.0
Mean chronological age at time of MRI-examination, weeks (SD)	14.7 (2.2)
Range of chronological age at time of MRI-examination, weeks	10.3–24.0
Mean post-menstrual age at time of MRI-examination, weeks (SD)	40.9 (1.7)
Range of post-menstrual age at time of MRI-examination, weeks	37.7–49.2

*MRI* magnetic resonance imaging, *SD* standard deviation

13.0% of the MRI examinations were considered of suboptimal quality, mostly due to motion artefacts.

Haemorrhage/blood products were seen in 60 (49.5%) of the neonates (25 male). There were no significant differences according to sex (*P*=0.31); thus, the results were pooled.

No SDHs or other extra-axial haemorrhages/blood products were identified.

All identified haemorrhages were located either in the germinal matrix, intraventricular and/or in the parenchyma. Isolated germinal matrix-intraventricular haemorrhages were most common (*n*=40, Fig. 2), with a small number of these having sequelae of porencephalic cyst (*n*=4, Fig. 3). Two infants had isolated cerebellar haemorrhages without germinal matrix haemorrhage (Fig. 4). A total of 14 infants had germinal matrix-intraventricular haemorrhages with cerebellar haemorrhages, of whom four also had sequelae of porencephalic cyst (Table 3).

**Table 3:** Localisation of intracranial haemorrhages in 121 ex-premature neonates as identified on magnetic resonance imaging around term-equivalent age

Localisation of intracranial haemorrhages	Number (% of total, *n*=121)
Intracranial haemorrhage	60 (49.5%)
Extra-axial haemorrhage	0 (0.0%)
Isolated germinal matrix-intraventricular haemorrhage, without sequelae of porencephalic cyst	40 (33.0%)
Isolated germinal matrix-intraventricular haemorrhage, with sequelae of porencephalic cyst	4 (3.3%)
Isolated cerebellar haemorrhage	2 (1.6%)
Germinal matrix-intraventricular haemorrhage, without sequelae of porencephalic cyst, with cerebellar haemorrhage	10 (8.3%)
Germinal matrix-intraventricular haemorrhage, with sequelae of porencephalic cyst, with cerebellar haemorrhage	4 (3.3%)

Intra-observer agreement for identification of haemorrhage/blood products was very good, with a kappa value of 0.90 (95% CI 0.82–0.98), while inter-observer agreement was good with a kappa value of 0.78 (95% CI 0.67–0.89).

The mean sinocortical width as measured on axial T2-weighted image was 3.5 mm (SD 1.4 mm, range 1.0–7.5 mm) and 3.3 mm (SD 1.2 mm, range 1.0–6.5 mm) on the right and left sides, respectively. The mean interhemispheric distance as measured on axial T2-weighted image was 3.1 mm (SD 1.1 mm, range 1.0–7.9 mm). Seventy-four infants (61.1%) had a sinocortical width > 3 mm on one or both sides (right, left, or both). Only two infants (1.6%) had an interhemispheric distance > 6 mm.

When measured on the ten coronal FLAIR sequences, the mean sinocortical width was 5.9 mm (SD 1.7) on the right side and 5.9 mm (SD 2.1) on the left side, while the mean interhemispheric distance was 3.6 mm (SD 1.6). In comparison, corresponding measurements obtained from axial T2 sequences in the same ten infants showed a mean sinocortical width of 2.7 mm (SD 1.2) on the right side and 3.3 mm (SD 1.1) on the left side, and a mean interhemispheric distance of 3.4 mm (SD 0.8).

## Discussion

In this study of 121 extremely premature infants around term-equivalent age, no SDH or other extra-axial haemorrhage or blood products were identified either supratentorially or infratentorially on T1-weighted, T2-weighted, or SWI sequences. Nearly half of the infants had markers of a previous intracerebral haemorrhage related to their prematurity, including isolated germinal matrix-intraventricular haemorrhages, germinal matrix-intraventricular haemorrhages with sequelae of porencephalic cysts, and cerebellar haemorrhages. Inter- and intra-observer agreement for identification of haemorrhage was good and very good, respectively. The mean sinocortical width was 3.4 mm (3.5 mm on the right side and 3.3 mm on the left side), whereas the mean interhemispheric distance was 3.1 mm, both measured on axial T2-weighted MRI. Seventy-four infants (61.1%) had a sinocortical width > 3 mm on one or both sides, while only two infants (1.6%) had an interhemispheric distance > 6 mm.

Very few studies have reported on the prevalence of SDH in infants born prematurely [14, 39]. In a study by Keenan and colleagues including 152 cases of serious or fatal inflicted traumatic head injury, the authors found a mildly increased odds ratio for inflicted brain injury and SDH in children born prematurely [39]. Hitherto, only one study by Högberg and colleagues, being a population-based register-study of 306 infants, has addressed infants born before 28 gestational weeks [14]. The study included all infants born between 1997 and 2014 diagnosed with SDH by using the ICD-10 diagnosis of either S06.5 (traumatic SDH) or I62.0 (nontraumatic SDH), with no mention of location, appearances, or method of identification. This clearly reduced the study’s potential to recognise birth-related posterior fossa SDHs. The overall incidence of SDH was 16.5 per 100,000 infants, and the median age was 2.5 months. For infants aged 7–365 days, acute nontraumatic SDH was associated with multiple birth, preterm birth, and small-for-gestational age. Infants born prior to gestational week 32 had odds ratios of 1.96 for traumatic SDH, 3.21 for nontraumatic SDH, and 4.17 for SDH and an abuse diagnosis. The authors conclude that the increased odds for acute nontraumatic SDH in preterm births or small-for-gestational age indicate a perinatal vulnerability for SDH beyond the first week of life. Our findings do not support this hypothesis, as we did not find any evidence of SDH around term-equivalent age in our group of 121 infants born before 28 gestational weeks. Högberg and colleagues acknowledged several limitations to their study though, including a lack of validation of the SDH-diagnoses. They state that they used “observation for suspected abuse”, “battered baby syndrome”, or “maltreatment syndrome” as definition of an abuse diagnosis, but they did not specify whether (or which) specific ICD-10 codes were used. Neither did they address whether it is common practice to set an ICD-10 abuse-diagnosis in Sweden in cases of suspected or confirmed abuse. We consider these to be additional limitations, as both the use and predictiveness of ICD-10 codes for child abuse have been shown to vary considerably in clinical practice [40]. Given that our findings do not support the conclusions made by Högberg and colleagues and considering the limitations of their study, their conclusions must be read with caution.

In our study, given the infants’ mean chronological age of 14.7 weeks at the time of the MRI examination, the appearances of a potential SDH would have differed according to its developmental stage and potential rebleeds and to the type of MRI contrast used (i.e. T1- or T2-weighted). Our protocol was designed to detect both acute, subacute, and chronic SDHs, as well as markers of a previous bleed (hemosiderin), using T1-weighted, T2-weighted, and SWI. However, and opposite to parenchymal brain haemorrhages, hemosiderin deposits may or may not be present after a SDH [41, 42]. It has been suggested that the absence of a blood–brain barrier in the subdural space allows for dilution and clearance of hemosiderin [41, 42], hampering the sensitivity of hemosiderin deposits as a marker of SDHs. In term infants, birth-related SDHs are relatively prevalent, with most resolving completely within 1–3 months [12, 13], and they rarely go on to form chronic SDHs [12, 13, 43]. Little has been published on infants born extremely premature regarding this. Assuming that the prevalence and development of SDHs in infants born extremely premature may resemble that of term-born infants, it is possible that some of the infants could have had small previous SDHs that were fully reabsorbed at the time of the MRI scan around term-equivalent age, with no remaining hemosiderin deposits.

Compared to infants born at term [20–22], a larger percentage of infants born prematurely have prominent subarachnoid spaces at term-equivalent age [23–25]. Infants born prematurely are at risk of various encephalopathies including periventricular leukomalacia, germinal matrix- and intraventricular haemorrhage, parenchymal venous haemorrhagic infarction, and post-haemorrhagic hydrocephalus [23, 26], which may result in brain atrophy and prominent subarachnoid spaces [24, 27, 28]. It has also been suggested that premature birth in itself might give reduced brain growth, resulting in prominent subarachnoid spaces [29]. In the setting of BESS, it has been suggested that the enlarged subarachnoid spaces predispose to the development of spontaneous SDHs or SDHs after minor head trauma [15–19] due to increased stretching of the bridging veins, making them more vulnerable to tearing and subsequent bleeding into the subdural space [44, 45]. This hypothesis has since been challenged by the results of Raul and colleagues, suggesting that the enlargement of the subarachnoid space may actually dampen the displacement of the brain relative to the skull during motion, thereby reducing the stretch on bridging veins during brain-motion within the skull [46]. The pathophysiology of prominent subarachnoid spaces in ex-prematures might be different from that of BESS. However, some similarities exist, such as a longer route for the traversing vessels with potential stretching, which has been demonstrated by a mathematical model [30]. Nevertheless, the association between a greater depth of the subarachnoid space and an increased risk of SDHs is controversial [15, 31].

Studies that provide population-based references of subarachnoid space measurements for both infants born at term and infants born prematurely typically rely on cranial coronal ultrasound measurements [20–22, 24, 29, 47, 48] rather than on axial MRI, as performed in our study. However, linear measurements of the subarachnoid space obtained from cranial ultrasound in the coronal view have shown a good correlation with measurements obtained from coronal MRI [25, 49]. Further, a recent study, including 63 infants from 4 days to 24 months of age, found that the width of the subarachnoid spaces was slightly larger when measured on coronal T2-weighted images as compared to axial T2-weighted images [50]. Although based on only a small subset of our cohort (*n* = 10) who underwent coronal FLAIR imaging, the measurements of the subarachnoid spaces obtained from coronal FLAIR sequences were larger than those obtained from axial T2-weighted images. Taken this into consideration, our reported values are rather conservative as the subarachnoid spaces probably would have been slightly wider if measured coronally.

In term newborns, the mean sinocortical width measured on cranial ultrasound has previously been reported from 1.2–2.0 mm [20–22], which is significantly lower than our mean sinocortical width of 3.4 mm. A sinocortical width of 3.0 mm and an interhemispheric distance of 6.0 mm have been proposed as upper limits to distinguish normal from pathologically dilated subarachnoid spaces in infants, as measured on coronal ultrasound [21]. In our study, 61.1% had a sinocortical width > 3.0 mm on one or both sides, and only 1.6% had an interhemispheric distance > 6.0 mm, as measured on axial MRI images. According to the aforementioned paper [50], our figures are most likely underestimating the coronal width; thus, a large percentage of the ex-premature infants in our study have prominent subarachnoid spaces as compared to infants born at term. Since increased width of the subarachnoid space represents an important marker of underlying pathology, we suggest that reference standards based on different imaging methods and planes might prove useful.

There is limited published data on the width of subarachnoid spaces in infants born prematurely, measured at term-equivalent age. However, Vo Van and colleagues evaluated the subarachnoid space dimensions (craniocortical width and interhemispheric distance) using both cerebral MRI and ultrasound at term-equivalent age in infants born prematurely before 32 weeks of gestation [25]. They found a mean craniocortical width measured on coronal MRI of 5.0 mm on the right side and 5.1 mm on the left side, and mean craniocortical width measured on ultrasound of 3.3 mm on the right side and 3.0 mm on the left side, respectively. The interhemispheric distance was reported at 4.8 mm measured on MRI and 4.1 mm measured on ultrasound. As the craniocortical width is generally considered to be slightly wider than the sinocortical width [21] and we measured sinocortical rather than craniocortical width, their measurements are not directly comparable to ours. However, their interhemispheric distance measurements are slightly larger than those observed in our study. Regarding BESS, there is currently no consensus on imaging criteria and no established cut-off values, but age-dependent sinocortical-, craniocortical-, and interhemispheric widths above the 95th percentiles are considered abnormal [51].

The strengths of this study are the relatively high number of examined infants, its population-based design, state-of-the-art MR imaging, the meticulous assessment of the images by highly experienced observers, and the high agreement between observers for diagnosing haemorrhage/blood products. We do acknowledge limitations to our study, however. We did not obtain clinically measured head circumferences at the time of MRI conducted around term-equivalent age, which would have been relevant for several reasons. These measurements could have provided valuable clinical-radiological correlations within this cohort and allowed for a clearer demonstration of differences between ex-premature infants with prominent subarachnoid spaces and those with BESS in future studies. This is particularly important because a significant percentage of the infants in our study had prominent subarachnoid spaces as measured on MRI. Due to the “feed-and-wrap” technique used, some of the MRI examinations were aborted because of an uneasy or moving child. Thus, the SWI sequence was unsuccessful in 23 infants, which might have led to missed small hemosiderin deposits based on T2-weighted images alone. A coronal FLAIR sequence was added in 10 patients only, and no other coronal sequences or reconstructions were performed, with the potential of missing subtle SDHs. In addition, 13.0% of the examinations were considered of suboptimal quality mostly due to motion artefacts. Nevertheless, no appreciable SDH could be detected around term-equivalent age with the performed sequences.

## Conclusion

When studied with MRI around term-equivalent age, 49.5% of extremely premature infants exhibited intracerebral haemorrhage with good/very good agreement, but no evidence of SDH. Our results do not lend support to the hypothesis that premature infants are more prone to SDH unrelated to AHT during the first 3–4 months of life. The sinocortical width exceeded 3.0 mm in 61.1% of infants.

## Data Availability

Data supporting this study are not publicly available due to their containing information that could compromise the privacy of research participants. Please contact the first author.
